# Physicochemical and Microbial Regulation Inhibit Rice Mercury Accumulation in the Karst Region with High Geological Background

**DOI:** 10.3390/toxics14070615

**Published:** 2026-07-15

**Authors:** Yanxin Hu, Zhengcheng Song, Lu Qiao, Xinyu Liang, Shaochen Yang, Junyao Yan, Langfei Wei, Jinjuan Li, Ping Li

**Affiliations:** 1 College of Resources and Environmental Engineering, Guizhou University, Guiyang 550025, China; huyanxin1102@126.com (Y.H.); gs.lqiao24@gzu.edu.cn (L.Q.); summy_lee@163.com (J.L.); 2 Laboratory of Karst Environmental Evolution and Ecological Security, Institute of Geochemistry, Chinese Academy of Sciences, Guiyang 550081, China; songzhengcheng@mail.gyig.ac.cn (Z.S.); liangxy98@163.com (X.L.); yangshaochen@mail.gyig.ac.cn (S.Y.); yanjunyao@mail.gyig.ac.cn (J.Y.); weilangfei@mail.gyig.ac.cn (L.W.); 3 University of Chinese Academy of Sciences, Beijing 100049, China

**Keywords:** mercury, high geological background, rice, remediation, microbial regulation

## Abstract

Methylmercury (MeHg) accumulation in rice is a major source of human MeHg exposure in some inland areas, yet the mechanism controlling mercury (Hg) accumulation in high geological background (HGB) regions remains poorly understood. Here, a field-scale remediation experiment was conducted in a karst HGB region of Guizhou, China, using a synergistic strategy combining selenium foliar spraying and calcium oxide-based soil conditioner. The combined treatment reduced total Hg and MeHg concentrations in rice grains by 63.0% and 80.0%, respectively. The root uptake from the soil–water system was found to be the primary pathway controlling Hg transfer into rice grain. Mechanistically, the soil conditioner increased soil pH and reduced bioavailable Hg, porewater Hg, and soil MeHg by 53.6%, 59.8%, and 62.6%, respectively, whereas selenium foliar spraying promoted Hg–Se complexation and reduced Hg mobility in the paddy system. In parallel, the combined treatment suppressed Hg-methylating microorganism (e.g., Geobacter decreased by 65%) and hgcAB gene (hgcA: −65%; hgcB: −58%), while enriching Hg-resistant taxa and merA-mediated detoxification pathway. These coupled physicochemical and microbial processes substantially reduced Hg bioavailability, MeHg production, and Hg bioaccumulation in rice grain of the paddy ecosystem, providing an effective strategy for mitigating Hg-related food safety risk in the karst HGB region.

## 1. Introduction

Mercury (Hg) is a globally pervasive toxic heavy metal. Once emitted into the atmosphere, Hg can undergo long-range atmospheric transport and deposition, thereby affecting the ecosystem on a global scale [1]. Following its release into the environment, Hg can be transformed into highly toxic methylmercury (MeHg) under anerobic conditions mediated by microbial activity [2]. MeHg poses a substantial threat to human health because of its ability to cross the placental barrier and its strong biomagnification through the aquatic and terrestrial food web [3]. Global economic losses associated with the adverse health effects of human MeHg exposure are estimated to reach approximately US$117 billion annually [4]. Rice consumption has been recognized as one of the major pathways of human MeHg exposure worldwide, particularly in mining regions and inland areas of China [5], yet the effectiveness of remediation strategies in high geological background areas remains unclear, motivating the present field experiment.

Rice grain can accumulate a substantial amount of MeHg from paddy soil [6]. Previous studies on Hg bioaccumulation mechanism in rice have primarily focused on mining areas characterized by extremely elevated Hg concentrations in both soil (up to 100 µg g^−1^) and the atmosphere (up to 80 ng m^−3^) [7,8,9]. For example, Hg isotope analysis demonstrated that different rice tissues assimilate Hg from soil and atmospheric sources in varying proportions [9]. Subsequent studies using Hg speciation isotopes further revealed two dominant pathways of Hg transfer into rice grain: inorganic Hg (IHg) is derived predominantly from atmospheric sources, whereas MeHg originates almost exclusively from the soil–water system [7]. Notably, rice leaf can directly absorb Hg from the atmosphere and subsequently translocate it to the grain, indicating that both total gaseous mercury (TGM) and paddy soil are major sources of IHg in rice plants [7].

Recent studies conducted in high geological background (HGB) areas (i.e., regions with naturally elevated trace element concentrations derived from geological substrates rather than anthropogenic inputs) of Guizhou, China, have revealed a distinct pattern of Hg bioaccumulation in rice. In karst regions, the highly developed underground fissure and conduit systems facilitate rapid element leaching and migration, while soil Hg speciation undergoes continuous transformation during pedogenesis [10]. Some paddies with low soil Hg concentration still result in high Hg accumulation in rice grain that exceeds the food safety limit. For instance, in Luodian County, Guizhou Province, the mean total Hg (THg) concentration in paddy soil is only 0.39 mg kg^−1^; nevertheless, 48.5% of polished rice samples exceed the permissible Hg limit (0.02 mg/kg) for food product [11]. The phenomenon of elevated Hg accumulation in rice from low-contamination soils is not unique to karst regions of China. Distinct geogenic Hg inheritance within surface soils has also been documented across the Mediterranean metallogenic zones [12], seasonally inundated floodplains of the Brazilian Amazon [13], and boreal agricultural soils of Northern Europe [14]. Despite these geographically widespread occurrences of HGB, the biogeochemical mechanisms governing Hg accumulation in rice from these regions remain poorly characterized, and effective remediation strategies tailored to HGB conditions are urgently needed. These observations suggest that the mechanism previously proposed to explain Hg accumulation in rice from mining regions may not be directly applicable to HGB areas. However, the biogeochemical processes governing Hg cycling and accumulation in these regions remain poorly understood, thereby limiting the development of effective Hg remediation and risk-control strategies.

To address these knowledge gaps, a field-scale synergistic remediation experiment was conducted in a typical karst HGB region of Guizhou Province, China. The specific objectives were: (i) to identify the dominant Hg uptake pathway into rice grain (root uptake vs. foliar absorption); and (ii) to test the efficacy of an integrated remediation strategy combining a calcium oxide-based soil conditioner with selenium foliar application. Furthermore, to elucidate the underlying mechanism, the novelty of our experiment lies in three aspects, and this study: (i) provides the first field-scale evidence in a karst HGB region that root uptake, rather than foliar absorption, is the dominant pathway controlling Hg transfer into rice grain; (ii) demonstrates that a synergistic strategy targeting both pathways achieves superior remediation efficacy; and (iii) couples multi-scale mineralogical characterization (FTIR, XRD, TEM-EDS) with microbial community profiling and functional gene quantification (hgcA, hgcB, merA) to reveal the coupled physicochemical and microbial regulatory mechanisms. This integrated approach moves beyond single-pathway remediation and provides a paradigm shift from the traditional “mining-area” model to the “HGB” model for rice Hg control. The results provided crucial data support for the regulation of rice Hg in the HGB region.

## 2. Materials and Methods

### 2.1. Study Area and Sample Collection

**Figure 1:**
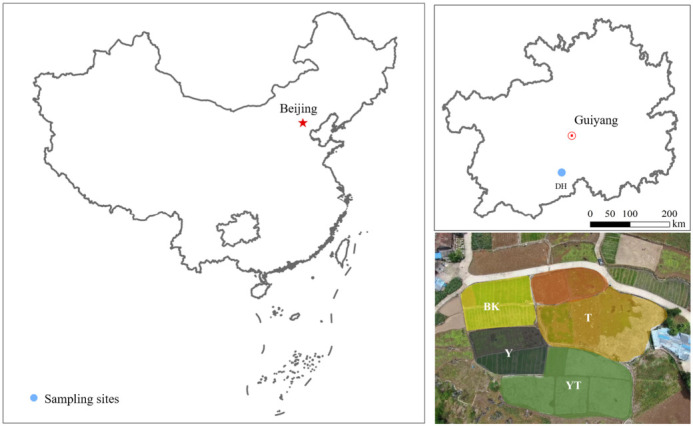
Geographical location of the study area.

The field experiment was conducted in DH Village (106°39′14″ E, 25°38′12″ N), Luodian County, Guizhou Province, China (Figure 1), which is characterized by a southern subtropical monsoon climate. The region has a mean annual temperature of 20.5 °C and an average annual precipitation of approximately 1200 mm. Elevations range from 400 to 600 m above sea level. The parent materials consist primarily of fluvial sediments and weathered carbonate rocks, resulting in the development of calcareous soil classified as Leptosol. The study area represents a typical karst region with a naturally high geological background and is free from mining activities within a 10 km radius.

The karst HGB region in Guizhou was selected because, despite soil Hg concentrations being only 0.39 mg kg^−1^ on average (far below those in mining areas), approximately 48.5% of rice grains exceed the permissible Hg limit (as mentioned above), suggesting that the widely accepted “mining-area” model for Hg accumulation could not be applied here. This unique contradiction makes this region a critical testbed for investigating the dominant pathways and developing targeted remediation strategies for HGB-affected agricultural systems.

The study area covered approximately 0.67 ha and was divided into four treatment blocks separated by 0.4 m-wide buffer strips. To prevent lateral movement of water and nutrients, waterproof membranes were installed along the buffer zones to a depth of 0.5 m. Four groups were established: (i) blank control (BK), consisting of conventional water and fertilizer management; (ii) foliar spray (Y), in which a selenium-containing foliar fertilizer was applied at the tillering and grain-filling stages at a rate of 7.5 L ha^−1^ per application; (iii) soil conditioner (T), involving application of a calcium oxide-based soil amendment with 3000 kg ha^−1^ at 30 days before rice transplanting, followed by incorporation into the upper 0–20 cm soil layer through rotary tillage; and (iv) combined group (YT), integrating both foliar spraying and soil amendment as described above. The rice cultivar used throughout the experiment was Taiyou 808. The application dosage of foliar selenium reagent was 22.5 kg per hectare, with a material cost of 107.64 USD per hectare. The dosage of calcium oxide soil conditioner was 1800 kg per hectare, corresponding to a cost of 579.60 USD per hectare. The combined treatment of selenium foliar spray and calcium oxide soil amendment required a total input of 1822.5 kg per hectare, with the total one-time material cost reaching 687.24 USD per hectare.

At physiological maturity, topsoil samples (0–20 cm) and corresponding whole rice plants were collected from each plot using a five-point sampling approach. In total, 57 soil samples and 57 rice plant samples were collected. Rice plants were further separated into root, stem, leaf, and grain. The number of samples for each treatment was as follows: BK (*n* = 12), Y (*n* = 11), T (*n* = 13), and YT (*n* = 21). Owing to the field-based nature of the experiment, samples were collected randomly and uniformly from each test area, which resulted in slightly unequal sample sizes across treatments. Soil samples were air-dried, manually cleared of gravel and plant debris, and sieved through a 2 mm nylon mesh. Rice tissues were thoroughly rinsed with deionized water, freeze-dried at −50 °C for 72 h, and ground into a homogeneous powder. All samples were sealed in polyethylene bags and stored at −80 °C prior to analysis.

### 2.2. Hg Measurement

THg concentrations in soil were determined by aqua regia digestion followed by cold vapor atomic absorption spectrometry (CVAAS; F732S, Shanghai Huaguang, Shanghai, China), with a method detection limit of 0.01 μg g^−1^. THg in plant tissues was measured using a direct Hg analyzer based on combustion gold amalgamation atomic absorption spectrometry (DMA-80, Milestone, Sorisole, Italy).

Porewater Hg concentrations were determined following USEPA Method 1631. Rhizosphere soils were collected between the grain-filling and maturity stages and centrifuged at 4000 rpm for 30 min to obtain porewater. The supernatant was acidified with 0.4% (*v*/*v*) HCl and stored in the dark at 4 °C prior to analysis. Hg concentrations were subsequently quantified by SnCl_2_ reduction–gold amalgamation cold vapor atomic fluorescence spectrometry (CVAFS; Brooks Rand Model III, Seattle, WA, USA).

A DTPA solution (0.005 mol L^−1^ DTPA, 0.01 mol L^−1^ CaCl_2_, 0.1 mol L^−1^ TEA, pH 7.3) was selected to extract bioavailable Hg from soil (Supplementary Text S1, Figure S1). And 5.0 g of soil was mixed with 25 mL of the extractant in a 50 mL centrifuge tube and then shaken at 200 rpm for 2 h at 25 °C. The suspensions were then centrifuged at 4000 rpm for 30 min, and the supernatants were collected for Hg determination using the same method as for porewater Hg measurement.

Methylmercury (MeHg) concentrations were determined following alkaline digestion and solvent extraction, coupled with gas chromatography–cold vapor atomic fluorescence spectrometry (GC–CVAFS; Brooks Rand Model III, Seattle, WA, USA).

### 2.3. Soil Microbial Community and Functional Genes

Total genomic DNA was extracted from 0.5 g of fresh soil using the PowerSoil^®^ DNA Isolation Kit (MoBio Laboratories, Carlsbad, CA, USA). DNA quality was assessed by 1% agarose gel electrophoresis and a NanoDrop 2000 spectrophotometer, and the purified DNA was stored at −20 °C. The V3–V4 hypervariable region of the bacterial 16S rRNA gene was amplified using primers 338F (5′-ACTCCTACGGGAGGCAGCA-3′) and 806R (5′-GGACTACHVGGGTWTCTAAT-3′). Amplicons were paired-end sequenced (2 × 250 bp) on an Illumina NovaSeq platform. Raw sequences were quality-filtered and denoised using QIIME2 (v2020.11) following the standard DADA2 pipeline, clustered into amplicon sequence variants (ASVs) at 100% similarity, and taxonomically assigned against the SILVA database (v138). Alpha diversity indices (Shannon, Chao1, Mothur 1.30.1) and community composition were calculated.

Absolute abundances of Hg-cycling functional genes (hgcA, hgcB, merA) were determined by real-time quantitative PCR (qPCR) on a QuantStudio 5 instrument (Applied Biosystems). Primers were taken from the published literature. Each 20 μL reaction contained SYBR Green Master Mix (10 μL), forward and reverse primers (0.4 μmol·L^−1^ each), and DNA template (2 μL). Thermal cycling conditions were: 95 °C for 10 min (initial denaturation); 40 cycles of 95 °C for 15 s, 60 °C for 30 s, and 72 °C for 30 s. Standard curves were generated from ten-fold serial dilutions (10^1^–10^7^ copies·μL^−1^) of plasmids containing the target gene inserts. Gene abundances are reported as copies per gram of dry soil (copies·g^−1^), and each sample was analyzed in three technical replicates.

### 2.4. Characterization of the Soil Conditioner

The mineralogical phase composition of the calcium-oxide-based conditioner was determined by X-ray diffraction (XRD, D8 Advance, Bruker, Karlsruhe, Germany) using Cu Kα radiation (λ = 1.5406 Å). Scans were performed over a 2θ range of 5–80° with a step size of 0.02° and a scanning speed of 2°·min^−1^. Surface functional groups were analyzed by Fourier transform infrared spectroscopy (FTIR, Nicolet iS50, Thermo Fisher Scientific, Waltham, MA, USA) using the KBr pellet method over a wavenumber range of 400–4000 cm^−1^ at a resolution of 4 cm^−1^, with 32 scans accumulated. Surface morphology and elemental signatures were examined by transmission electron microscopy (TEM, Tecnai G2 F20 S-Twin, FEI/Thermo Fisher Scientific, Hillsboro, OR, USA) with a point resolution of 0.24 nm and a lattice resolution of 0.102 nm.

Soil pH measurement and the experimental quality control of this study are presented in the Support Information file (Supplementary Texts S2 and S3).

## 3. Results and Discussion

### 3.1. Mitigation of Hg Accumulation in Rice Grain

**Figure 2:**
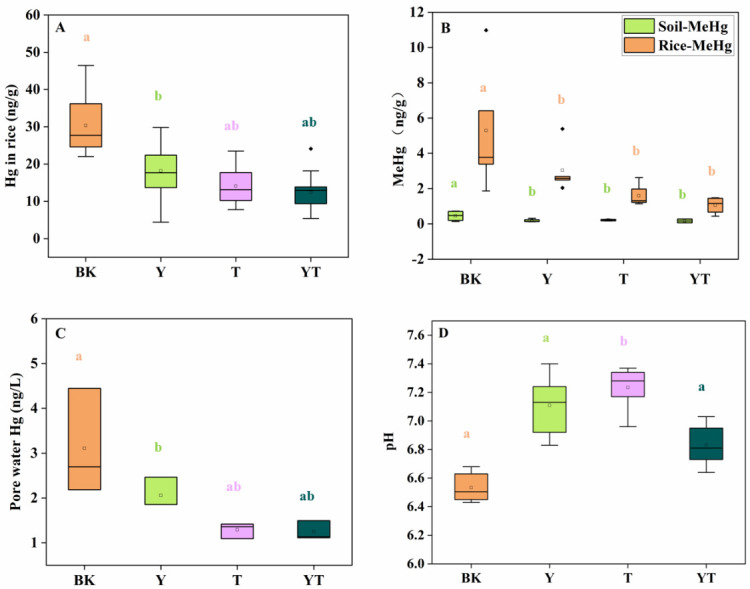
Comparison of relevant indicators between different groups. (**A**) Boxplot comparing Hg content in rice from different groups using unprocessed baseline data. (**B**) Bar chart showing MeHg content in grains and soil MeHg content under different treatments. (**C**) Boxplot illustrating differences in Hg content in pore water under different treatments after repair. (**D**) Boxplot of soil pH changes under different treatments (BK, Y, T, YT). Yellow represents Group BK, green represents Group Y, pink represents Group T, and dark green represents Group YT. Letters a, b and ab indicate significant differences. The same letter means no significant difference, whereas different letters mean a significant difference exists.

The synergistic remediation strategy exhibited a pronounced inhibitory effect on Hg accumulation in rice grains. THg concentrations in rice grains displayed a significant decreasing gradient among the three treatment groups (Figure 2A). Grain THg concentrations were 28.9 ± 9.6 ng g^−1^ in the BK group (*n* = 12), 15.8 ± 5.2 ng g^−1^ in the Y group (*n* = 11), 12.4 ± 4.1 ng g^−1^ in the T group (*n* = 13), and 10.7 ± 3.5 ng g^−1^ in the YT group (*n* = 21), respectively. Compared to the BK group, grain THg concentrations in the Y, T, and YT groups were reduced by 45.3%, 57.1%, and 63.0%, respectively, demonstrating significant effects on Hg mitigation. High reductions were also observed for rice grain MeHg concentrations (Figure 2B). The BK group exhibited a grain MeHg concentration of 5.29 ± 3.58 ng g^−1^ (*n* = 5), whereas MeHg concentrations decreased to 3.04 ± 1.34 ng g^−1^ in the Y group, corresponding to a 42.5% reduction. Grain MeHg concentrations in the T group were further reduced to 1.60 ± 0.69 ng g^−1^, representing a 69.8% decrease. The YT group achieved the strongest inhibitory effect, with a grain MeHg concentration of 1.06 ± 0.49 ng g^−1^ and an overall reduction of 80.0%. These results demonstrated that simultaneous regulation of both foliar uptake and root uptake pathways can substantially suppress Hg accumulation in rice grains. Among the three remediation treatments, the effectiveness of Hg mitigation followed the order: YT group > T group > Y group. The mechanisms underlying inhibition along these distinct uptake pathways are discussed below.

The MeHg mitigation efficacy achieved in this study compares favorably with a range of previously reported remediation strategies for reducing MeHg accumulation in rice grains (Table 1). For instance, co-amendment with low-rate biochar and selenium reduced grain MeHg concentrations by 29.1–91.6% in slightly Hg-impacted paddy soils [15]. Pot trials applying 0.1–1 wt% thiol-modified montmorillonite reduced grain MeHg by 43.9–62.3% [16]. Selenium- and chitosan-functionalized biochar decreased seed MeHg by 86.37% and 75.50%, respectively, via enrichment of Hg-tolerant rhizosphere Bacillus that suppresses soil MeHg biosynthesis [17]. Collectively, the 80.0% reduction in grain MeHg achieved by our combined selenium foliar spray and calcium oxide-based soil conditioner ranks among the highest reported efficiencies for paddy rice MeHg mitigation. Notably, whereas many of these studies were conducted in Hg mining areas with elevated soil Hg concentrations or under controlled pot conditions, our field experiment was performed in a karst high geological background region with relatively low soil Hg levels (0.39 mg kg^−1^), demonstrating that the synergistic strategy is highly effective even under moderate Hg contamination scenarios. This highlights the broad applicability of our approach for mitigating MeHg-related food safety risks across diverse paddy systems.

### 3.2. Mechanism of Foliar Spraying

As discussed above, the foliar spray treatment effectively reduced Hg accumulation in rice grain. The mitigation mechanism of this pathway was proposed to involve two complementary processes. First, rice leaf can directly absorb atmospheric Hg through stomata, after which Hg is translocated to the rice grain [9]. In the foliar spray treatment, foliar-applied Se may establish a physiological barrier within plant tissues. Specifically, foliar-absorbed Se and atmospheric-derived Hg can co-precipitate in the cytoplasm as insoluble Hg–Se nanoparticles (e.g., HgSe) [18], which may subsequently be sequestered within vacuoles or bound to cell walls. In addition, Se can enhance the expression of thiol metabolism-related genes, thereby limiting Hg uptake and translocation within plant tissues [15,18], a process that parallels the generalized heavy metal detoxification mechanisms involving antioxidant enzyme activation and metal chelation reported in other plant species [19]. Nevertheless, it should be noted that this direct foliar barrier effect represents a short-term, and aboveground intervention does not address the primary source of MeHg in the soil–root system, where the majority of Hg entering the grain via root uptake [9].

However, this mechanism is suggested to be unlikely to represent the dominant pathway responsible for Hg mitigation in the study area. Atmospheric Hg concentrations at the study site were relatively low (1.2 ± 0.1 ng m^−3^, *n* = 4), as measured using carbon traps. These levels are substantially lower than those reported for Hg mining regions, where atmospheric Hg concentrations can reach up to 80 ng m^−3^ [8]. Under such highly contaminated conditions, previous studies demonstrated that most inorganic Hg in rice grains originates from atmospheric uptake by rice foliage [7]. In contrast, the comparatively low atmospheric Hg concentrations observed in this karst region suggest that foliar Hg uptake contributes only marginally to grain Hg accumulation. Therefore, the rice leaf in the study area was proposed to possess a limited capacity for atmospheric Hg accumulation and subsequent translocation to rice grain.

On the other hand, because foliar spraying was conducted under open-field conditions, a portion of the selenium-containing solution inevitably entered the soil–paddy water system. Therefore, the foliar-applied Se may indirectly alter soil geochemical conditions and consequently influence Hg accumulation in rice through the soil uptake pathway. This can be demonstrated by the changes in soil bioavailable Hg and MeHg levels. Compared with the BK group, the Y group reduced DTPA-extractable Hg and soil MeHg concentrations by 14.0% and 59.0%, respectively, relative to concentrations of 19.2 ± 5.87 ng L^−1^ and 0.186 ± 0.092 ng g^−1^ in the BK group (*n* = 4). In addition, porewater Hg concentrations decreased by 33.8% compared with the BK group (3.11 ± 1.19 ng L^−1^, *n* = 3) (Figure 2C). These results indicate that foliar application altered Hg geochemical behavior within the paddy soil–water system.

This effect is likely attributable to the formation of poorly soluble HgSe complexes through the interaction between Se and Hg [20,21], which decreases Hg bioavailability in paddy soil. Reduced Hg bioavailability can subsequently suppress microbial MeHg production and limit Hg uptake by rice root and stem, thereby lowering both THg and MeHg concentrations in rice grain [22]. This indirect soil-mediated pathway is mechanistically more effective because it targets the source of Hg bioavailability in the rhizosphere, whereas the direct foliar barrier merely intercepts a minor atmospheric contribution. Collectively, these findings suggest that, under the relatively low atmospheric Hg condition of the study area, foliar uptake is not the dominant pathway controlling Hg accumulation in rice grain. Instead, root uptake from the paddy soil–water system appears to be the primary pathway governing Hg accumulation in rice grain. This distinction is important because it explains that the combined YT treatment, which simultaneously targets both foliar and root pathways, achieved the highest mitigation efficacy among all treatments.

### 3.3. Mechanism of Soil Amendment

The soil conditioner mitigated Hg accumulation primarily by reducing Hg bioavailability within the rhizosphere soil. Relative to the BK group, soil MeHg concentrations, porewater Hg concentrations, and DTPA-extractable Hg in the T group decreased by 52.2%, 58.5%, and 48.7%, respectively. In parallel, soil pH increased from 6.53 ± 0.10 in the BK group to 7.23 ± 0.15 in the T group (Figure 2D). These results demonstrate that the calcium oxide-based soil amendment substantially altered soil geochemical conditions and Hg speciation, thereby suppressing the accumulation of both THg and MeHg in rice grain.

**Figure 3:**
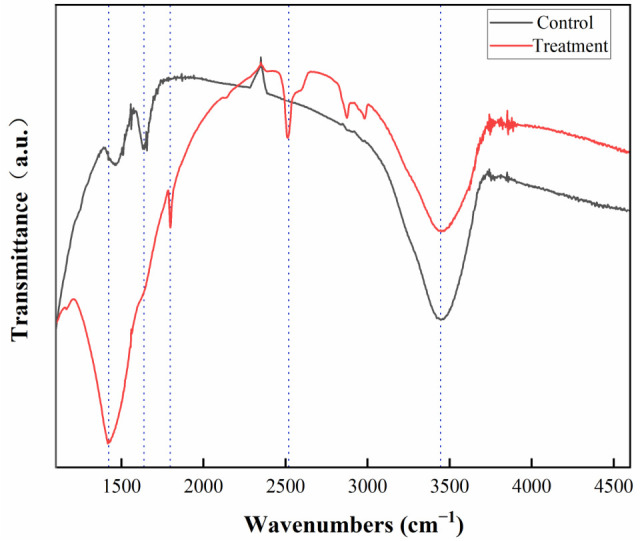
Fourier-transform infrared (FTIR) spectra of the soil conditioner. Control group is the infrared spectrum before adding the conditioner; treatment is the spectrum of the soil after YT treatment. The dashed lines mark the characteristic peaks, indicating different functional groups.

To elucidate the immobilization mechanism of the calcium oxide-based conditioner in karst paddy soil, the changes in the chemical coordination environment, mineralogical host phases, and microscale interfacial structures before and after amendment application were systematically characterized using Fourier-transform infrared spectroscopy (FTIR) (Figure 3), X-ray diffraction (XRD) (Figure S2), and transmission electron microscopy coupled with energy-dispersive spectroscopy (TEM–EDS) (Figure S3).

FTIR analysis provided molecular-scale evidence for Hg immobilization through functional group interactions. Spectral shifts indicated carboxyl group chelation, deprotonation of phenolic –OH groups, and π-complexation with aromatic structures, suggesting the formation of organic ligand-bound Hg species in soil [23,24]. Building upon these observations, XRD analysis revealed mineral-scale processes associated with Hg stabilization. The soil amendment hydrated to form Ca(OH)_2_ and Mg(OH)_2_, increasing soil pH from approximately 6.5 to >7.2 and promoting Hg^2+^ hydrolysis. No crystalline Hg-bearing minerals, such as HgO or HgS, were detected, indicating that Hg was predominantly retained as amorphous surface complexes rather than discrete mineral precipitates [25]. Combined FTIR and XRD results suggest that Hg was immobilized mainly through inner-sphere surface complexation, including ≡Ca–O–Hg^+^ and ≡CO_3_–Hg^+^ configurations [26,27]. Importantly, this mechanism avoids the introduction of exogenous sulfur phases that may otherwise stimulate Hg methylation.

TEM–EDS further provided microscale visualization of the evolving reactive interface. Following the amendment application, the conditioner surface was coated with Fe oxide, clay mineral, and organic matter. This process likely resulted from localized alkalization induced by the conditioner, which promoted dissolution of native Fe oxide in soil. The released Fe^3+^ ion subsequently hydrolyzed to form nanoscale hydrous Fe oxides [28], which possess abundant Fe–OH reactive sites capable of forming strong inner-sphere complexes with Hg [29,30]. Unlike many sorbent materials whose adsorption capacity decreases following surface coating [31], the formation of this composite interfacial layer appears to enhance Hg immobilization over time, thereby explaining the sustained remediation performance observed over multiple years [32]. Collectively, the three analytical approaches establish a coherent hierarchical framework in which FTIR identifies molecular-scale binding mechanisms, XRD constrains mineral-scale amorphous complexation, and TEM–EDS visualizes the dynamic evolution of the reactive composite interface.

Overall, these findings indicate that the calcium oxide-based soil conditioner transforms labile Hg^2+^ into low-bioavailability surface-bound species through a coupled multi-scale immobilization mechanism: (i) molecular-scale functional group chelation and π-complexation revealed by FTIR; (ii) mineral-scale alkalinity-driven amorphous surface complexation identified by XRD; and (iii) microscale formation of Fe oxide-mediated composite interfaces observed by TEM–EDS. This hierarchical immobilization framework provides mechanistic insight into the effective control of Hg contamination in paddy soils within HGB karst regions characterized by naturally elevated Ca and Fe contents.

These results can explain that the combined treatment (YT) exhibited the strongest mitigation effect on Hg accumulation in rice grain. The YT group integrated the Hg-complexation capacities of both the selenium foliar spray and the calcium oxide-based soil conditioner. Relative to the BK group, soil MeHg concentrations, porewater Hg concentrations, and DTPA-extractable Hg in the YT group decreased by 62.6%, 59.8%, and 53.6%, respectively. These findings demonstrate that the synergistic dual-complexation of the combined treatment most effectively reduced the pool of bioavailable Hg in the soil–water system, thereby suppressing Hg transport and accumulation in the rice plant.

### 3.4. Microbial Regulation

**Figure 4:**
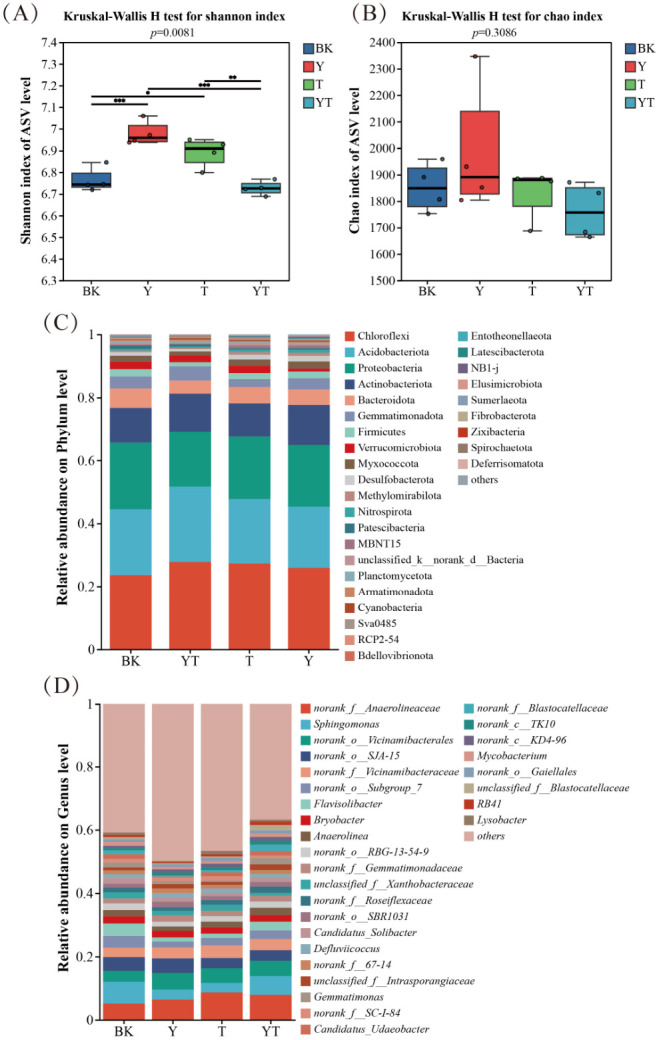
Analysis of microbial diversity and community structure (phylum/genus level) across different groups. (**A**) Shannon index of different treatment groups (BK, Y, T, YT). Significant differences between groups were marked with asterisks, where * represents *p* < 0.05, ** represents *p* < 0.01, and *** represents *p* < 0.001; (**B**) Chao index of different treatment groups; (**C**) stacked bar chart of relative abundance of microorganisms at the phylum level; (**D**) stacked bar chart of relative abundance of microorganisms at the genus level.

The remediation treatments not only altered the physicochemical properties of paddy soils but also markedly shaped the rhizosphere microbial community structure. Among the treatments, the YT group induced the most pronounced restructuring of the microbial community, characterized by a transition from enhanced microbial diversity to functional community reorganization. Compared with the control and single-treatment groups, the YT treatment significantly increased both the Shannon and Chao1 diversity indices (*p* < 0.01) (Figure 4A,B), representing a 25% increase in the Shannon index. These results suggest that the combined application of foliar Se and soil conditioner alleviated environmental stress and promoted microbial diversity in karst paddy soil. This finding is consistent with previous reports that Se amendment can enhance soil microbial diversity by reducing heavy metal stress [33].

At the phylum level (Figure 4C), the YT group enriched Proteobacteria and Actinobacteriota, two microbial groups commonly associated with Hg resistance, Hg reduction, and Hg immobilization processes, while substantially suppressing Chloroflexi, a phylum closely related to Hg methylation activity. More importantly, several potential Hg-methylating genera, including norank_f__Anaerolineaceae and Geobacter, showed marked declines in the YT group, with the relative abundance of Geobacter decreasing by 65% (Figure 4D). In contrast, Hg-resistant genera such as Sphingomonas and Gemmatimonas were significantly enriched, with Sphingomonas increasing by 3.5-fold relative to the control group. Similar shifts in microbial community composition, characterized by suppression of Hg-methylating taxa and enrichment of Hg-resistant populations, have been observed in Se-amended paddy soils [17].

These community shifts, when linked to the concurrent geochemical changes (i.e., elevated pH and reduced Hg bioavailability), suggested that the combined effects of soil conditioner and Se imposed strong selective pressures on the rhizosphere microbiome—suppressing anerobic Hg-methylating microorganisms while promoting Hg-resistant and detoxification-associated taxa. Consequently, the rhizosphere microbial community in the YT group evolved from a state of “diversity recovery” toward one of “functional guild restructuring,” establishing an ecological basis for the observed reduction in Hg bioavailability and subsequent decrease in Hg accumulation in rice grain. The observed shifts in microbial community composition and associated plant defense responses are consistent with findings from studies on heavy metal-tolerant plant–microbiome systems, where rhizosphere restructuring enhances host resistance to metal stress [34].

**Figure 5:**
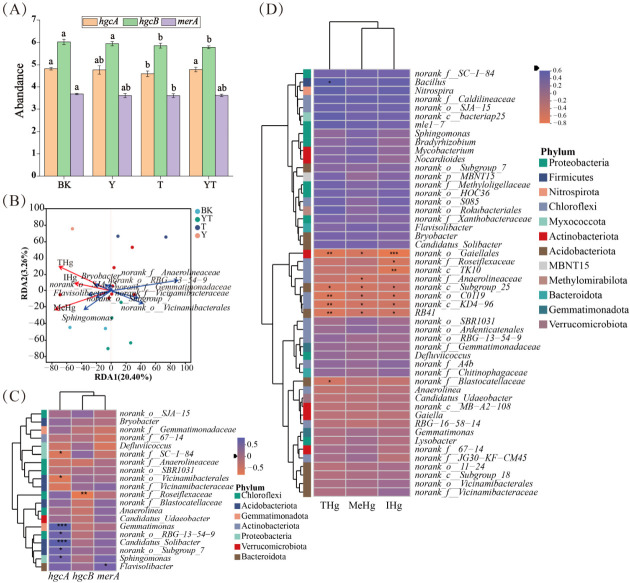
Comprehensive diagram of correlation between microbial communities and environmental factors and RDA. (**A**) Analysis of differences in three gene contents in soil among different treatment groups. Letters a, b and ab indicate significant differences. The same letter means no significant difference, whereas different letters mean a significant difference exists; (**B**) RDA of microbial communities at the genus level; (**C**) Spearman correlation heatmap between dominant genera at the genus level and three different genes; (**D**) Spearman correlation heatmap between phylum-level microorganisms, dominant groups at the genus level, and different Hg forms in rice. Significant differences between groups were marked with asterisks, where * represents *p* < 0.05, ** represents *p* < 0.01, and *** represents *p* < 0.001.

Consistent with the observed microbial community restructuring, the distribution of Hg-cycling functional genes further revealed a directional transition from Hg methylation toward Hg reduction and demethylation under the YT treatment. The abundances of the key Hg methylation genes hgcA and hgcB decreased by 65% and 58%, respectively, whereas the abundance of the Hg reduction and demethylation gene merA increased by 42% (Figure 5A). Together, these changes formed a characteristic regulatory pattern of “suppressed methylation and enhanced detoxification”. This pattern aligns with recent findings that Se amendment and elevated pH can reshape soil microbial communities toward reduced Hg methylation potential [35].

Redundancy analysis (RDA) further demonstrated that the YT group clustered the closest to the merA vector and farthest from Hg-methylating genera and Hg species (Figure 5B), suggesting that the YT group shifted the microbial community toward a Hg transformation pathway, potentially favoring Hg reduction and detoxification. Correlation analysis showed that hgcA and hgcB were significantly positively associated with potential Hg-methylating taxa, including norank_f__Anaerolineaceae and Anaerolinea, whereas merA exhibited strong negative correlations with these taxa and positive associations with Hg-resistant genera such as Sphingomonas (Figure 5C). Furthermore, microbial–Hg correlation analysis revealed that Geobacter and Anaerolinea were strongly positively correlated with soil MeHg concentrations, while Sphingomonas and Pseudomonas showed negative correlations with both soil THg and MeHg concentrations (Figure 5D).

Taken together, these findings demonstrated a linked response cascade under the YT treatment: suppression of Hg-methylating taxa (e.g., Geobacter) and hgcAB genes reduced MeHg production, while enrichment of Hg-resistant taxa and merA upregulation enhanced Hg^2+^ reduction and detoxification. These coupled microbial processes, together with the physicochemical immobilization discussed in previous sections, decreased the pool of labile Hg in the rhizosphere soil and ultimately reduced Hg accumulation in rice grain. Although functional gene abundance does not directly represent in situ transformation rates, the consistent shifts in microbial composition, Hg-cycling genes, and Hg speciation collectively support the proposed microbial regulation mechanism.

## 4. Conclusions

This study provided the first field-scale evidence that root uptake from the soil–water system, rather than foliar absorption, dominated rice Hg accumulation in karst regions with high geological background—a mechanism fundamentally distinct from that reported in Hg mining areas. This phenomenon was fundamentally different from the mechanism reported for Hg mining areas. The combined remediation strategy integrating selenium foliar spraying and calcium oxide-based soil conditioner effectively reduced THg and MeHg concentrations in rice grains by 63.0% and 80.0%, respectively.

Mechanistically, the calcium oxide-based conditioner decreased Hg bioavailability through pH elevation and multi-scale Hg immobilization, while selenium application promoted Hg–Se complexation and further reduced Hg mobility. In parallel, the combined treatment reshaped the rhizosphere soil microbial community by suppressing Hg-methylating microorganism (e.g., Geobacter, −65%) and hgcAB genes (hgcA: −65%; hgcB: −58%), while enriching Hg-resistant taxa and enhancing merA-mediated Hg detoxification (+42%). These coupled physicochemical and microbial processes collectively reduced labile Hg and MeHg production in paddy soil, thereby suppressing Hg accumulation in rice grain.

Overall, this study establishes a coupled “Hg immobilization and microbial regulation” framework for controlling rice Hg accumulation in the karst region with HGB. The proposed synergistic remediation strategy provides an effective and scalable approach for mitigating Hg-related food safety risk in an agricultural system with HGB. Looking forward, the applicability of this framework to other HGB regions worldwide warrants further investigation. Future research should prioritize (i) comparative studies across different HGB regions to validate the generalizability of our findings, (ii) long-term monitoring of remediation durability under diverse environmental conditions, and (iii) integration of this remediation approach with broader agricultural management practices to ensure both food safety and sustainable crop production. Such efforts will be essential for translating our mechanistic understanding into practical solutions for global Hg contamination challenges in HGB agricultural systems.

## Figures and Tables

**Table 1:** Comparison of remediation strategies for reducing MeHg accumulation in rice grains.

Remediation Strategy	Study Type	Soil THg	Grain MeHg Reduction	Reference
Biochar + Se co-amendment	Field (slightly Hg-impacted)	~0.5 mg kg^−1^	29.1–91.6%	[15]
Thiol-modified montmorillonite (0.1–1 wt%)	Pot (mining soil)	Elevated (Hg mining area)	43.9–62.3%	[16]
Se- or chitosan-functionalized biochar	Pot (spiked soil)	Artificially contaminated	75.5–86.4%	[17]
Se foliar spray + CaO-based soil conditioner (this study)	Field (karst HGB region)	~0.39 mg kg^−1^	80.0%	This study

## Data Availability

The raw data supporting the conclusions of this article will be made available by the authors on request.

## Supplementary Materials

The following supporting information can be downloaded at: https://www.mdpi.com/article/10.3390/toxics14070615/s1, Text S1: Comparison of various method for extraction bioavailable Hg in soil; Text S2: Soil pH measurement; Text S3: Quality control; Figure S1: Relation between bioavailable mercury content in different extractants of the demonstration zone and rice THg concentrations; Figure S2: X-ray diffraction (XRD) pattern of conditioner; Figure S3: TEM morphology and EDS elemental composition analysis of the conditioner.
