# Supplementary material for: Physicochemical and Microbial Regulation Inhibit Rice Mercury Accumulation in the Karst Region with High Geological Background
Source: Toxics. 2026 Jul 15;14(7):615. doi: 10.3390/toxics14070615 (PMC13418583; doi:10.3390/toxics14070615)
Supplement: Supplementary file 1 [file toxics-14-00615-s001.zip › toxics-4410568-supplementary.pdf]

Supplementary Material

Physicochemical and microbial regulation inhibit rice mercury accumulation in karst high geological background region

Yanxin Hu[1,2], Zhengcheng Song[2], Lu Qiao[1,2], Xinyu Liang[2,3], Shaochen Yang[2], Junyao Yan[2], LangFei Wei[2,3], Jinjuan Li[1], Ping Li[2,*]

[1]College of Resources and Environmental Engineering, Guizhou University, Guiyang, 550025, China

[2]Laboratory of Karst Environmental Evolution and Ecological Security, Institute of Geochemistry, Chinese Academy of Sciences, Guiyang, 550081, China

[3]University of Chinese Academy of Sciences, Beijing, 100049, China

Corresponding Author:

Ping Li, Fax: +86 851 85891609, Email: liping@mail.gyig.ac.cn

The file includes:

6 pages

Text S1 to S3

Figs. S1 to S3

Text S1: Comparison of various method for extraction bioavailable Hg in soil

To evaluate bioavailable Hg fractions in soil, we used four extractants: 0.01 mol L^{-1} $CaCl_2$, 1.0 mol L^{-1} NH_4OAc, a DTPA solution (0.005 mol L^{-1} DTPA + 0.01 mol L^{-1} $CaCl_2$ + 0.1 mol L^{-1} TEA, pH 7.3), and 0.1 mol L^{-1} HCl. Among these, the DTPA extraction gave a correlation coefficient of r = 0.54 between bioavailable Hg and rice Hg concentrations, which was higher than those for $CaCl_2$ (r = 0.25), NH_4OAc (r = 0.48), and HCl (r = 0.49) (Fig. S1). These results indicate that the DTPA method provides the most representative estimate of bioavailable Hg in soil in the study area.

Text S2: Soil pH measurement

Soil pH was determined potentiometrically. Briefly, 10.0 g of air-dried soil (<2 mm) was transferred into a 50 mL centrifuge tube and mixed with 25 mL of ultrapure water at a soil-to-water ratio of 1:2.5 (w/v). The suspension was shaken for 10 min and allowed to equilibrate for 30 min prior to measurement. The pH of the supernatant was measured using a portable pH meter (PHS-3C, LeiCi, Shanghai, China). All samples were analyzed in triplicate, and the results are reported as mean ± standard deviation (SD).

Text S3: Quality control

All used reagents were analytical grade or higher; experimental water was deionized to a resistivity of ⩾18.2 MΩ·cm. Glassware was combusted at 500 °C for 4 h, and used acids were ultrapure grade. Quality control for total Hg analysis included

soil CRM GBW07405 (determined value 288.2± 11.8 ng g^{-1}, n=11; reference value 290±40 ng g^{-1}; recovery rate 99.4%), citrus leaves CRM GBW10020 (determined value 142± 6.3 ng g^{-1}, n=11; reference value 150±20 ng g^{-1}; recovery rate 94.7%). MeHg quality control was performed with TORT-3 (determined value 132.9± 19 ng g^{-1}, n=6; reference value 137± 12 ng g^{-1}; recovery rate 96.9%). For each batch, blank, CRMs, and duplicate analysis were included. The standard deviation of three repeated measurements of the same sample was ≤10%, and the RSD of randomly selected duplicates was <6%. Raw sequencing data were quality-filtered, denoised, and clustered into ASVs at 100% similarity using QIIME2 as described above.

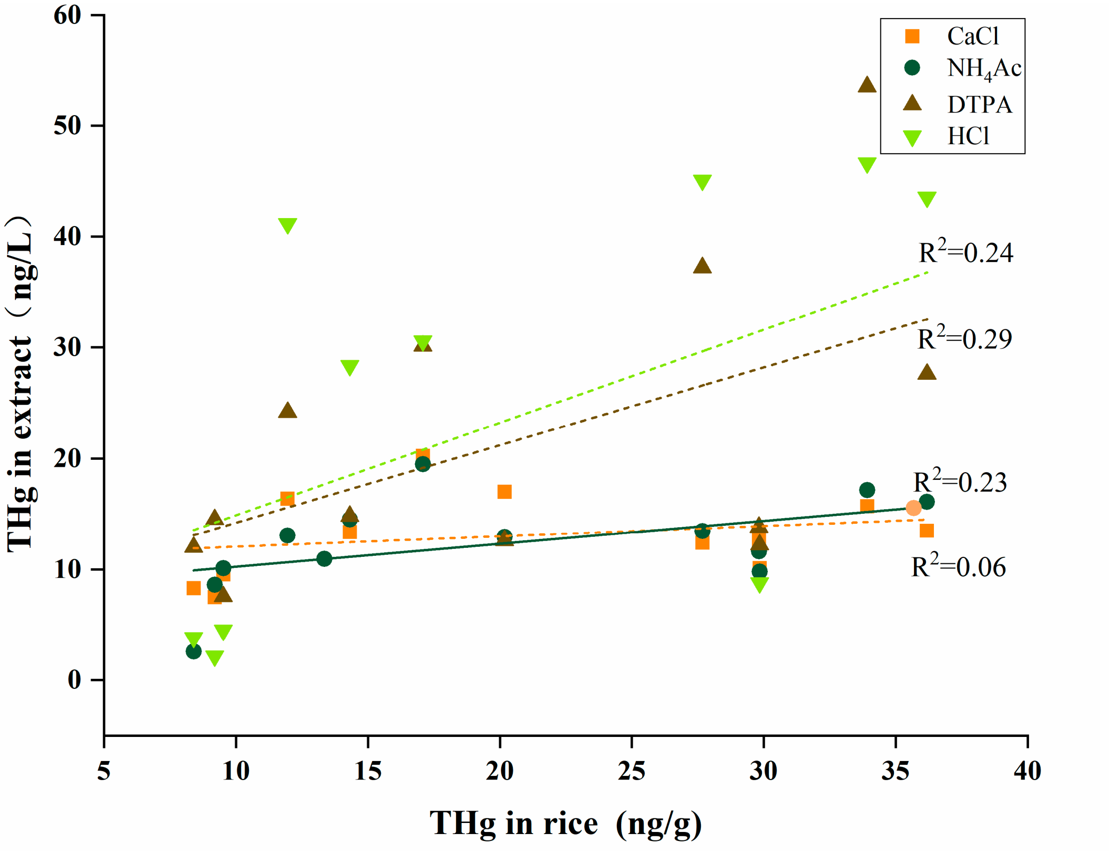

Figure S1. Relation between bioavailable mercury content in different extractants of the demonstration zone and rice THg concentrations.

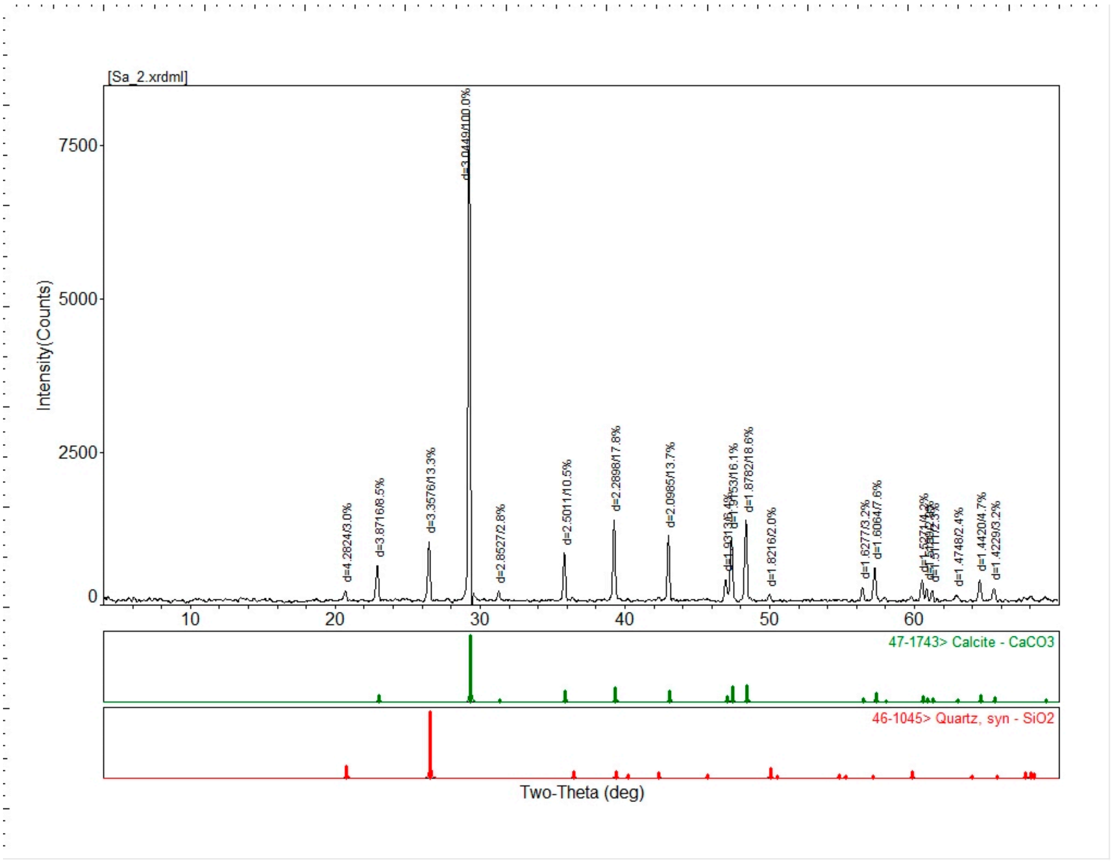

Figure S2. X-ray diffraction (XRD) pattern of conditioner.

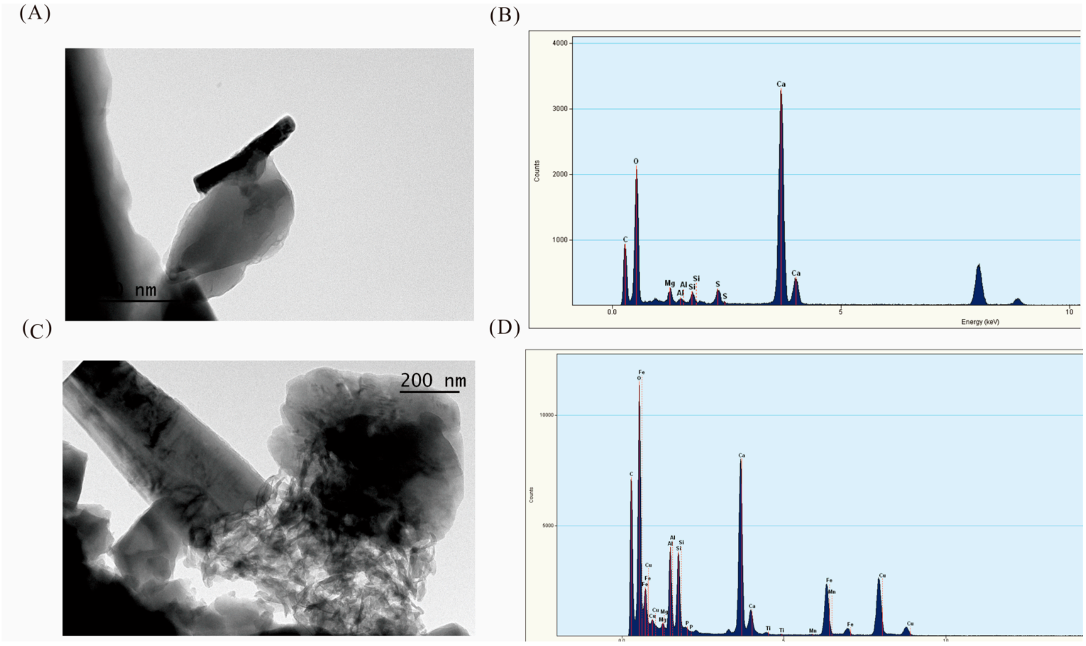

Figure S3. TEM morphology and EDS elemental composition analysis of the conditioner.

A: Original TEM morphology of the conditioner; B: original EDS analysis of the conditioner; C: TEM morphology of the conditioner isolated from paddy fields; D: EDS analysis of the conditioner isolated from paddy fields.
